# Is It Mum or the Dad? Parental Factors That Influence the Use and Acceptance of Fluoride Varnish among Parents in Eastern Saudi Arabia

**DOI:** 10.1155/2022/9109967

**Published:** 2022-12-13

**Authors:** Abdullah Aljami, Turki Alshehri, Abdulrahman Altuwaijri, Alhanoof Aldossary, Wejdan Almutairi, Faraz Ahmed Farooqi, Balgis Gaffar

**Affiliations:** ^1^College of Dentistry, Imam Abdulrahman bin Faisal University, Dammam, Saudi Arabia; ^2^Department of Dental Education, College of Dentistry, Imam Abdulrahman bin Faisal University, Dammam, Saudi Arabia; ^3^Department of Preventive Dental Sciences, College of Dentistry, Imam Abdulrahman bin Faisal University, Dammam, Saudi Arabia

## Abstract

**Objective:**

To investigate parental factors that influence knowledge, use, and acceptance of fluoride varnish (FLV) application.

**Methods:**

This cross-sectional study was conducted during December 2021. A multistage sampling technique was used to recruit parents with children aged 3 to 6 years and residing in Eastern Saudi Arabia, excluding those working in the dental field. The questionnaire consisted of four parts: demographics, knowledge, previous experience, and acceptance of FLV and was pilot-tested and distributed online using social media.

**Results:**

A total of 623 parents responded to the survey. Only 36.9% of parents had good FLV knowledge with dentists being the main source of information. Gender, educational level, family income, being in the medical field, and source of knowledge were factors that significantly (*P* < 0.05) influenced parental knowledge. Only 24.6% of the parents applied fluoride varnish to their children before, of whom only 29% were satisfied with the experience. Parent's FLV knowledge, view of dental visits, satisfaction with previous experience, perception of children's oral health, and willingness to reapply FLV were factors that significantly (*P* < 0.05) influenced FLV use. Working in the medical field, perception of dental visits, and the source of knowledge were factors that significantly (*P* < 0.05) influenced the parent's acceptance to apply FLV. While lack of knowledge about fluoride benefits (15%) was the main reason for parental refusal to apply FLV.

**Conclusions:**

The current study highlights the lack of knowledge and application of FLV among parents. There were more factors influencing mothers' acceptance of FLV application compared to fathers. Age, educational level, working in the medical field, perception, and patterns of dental visits were some of the identified factors. Dentists played a major role in parental knowledge and FLV acceptance. In a country with high caries prevalence, preventive programs such as FLV education and application are crucial.

## 1. Introduction

Dental caries is a multifactorial disease resulting from demineralization of the hard tooth structure and is the most common childhood disease worldwide [1, 2]. According to the World Health Organization (WHO), 60 percent to 90 percent of all school-aged children have dental caries [2, 3]. Higher caries prevalence among children was reported in Asian countries and regions such as China (84%), Korea (60.9%), Taiwan (81%), Thailand (77.7%), and Indonesia (90%), while lower prevalence was observed in Western countries such as Germany (26.2%) and UK (27.9%) [4]. Caries prevalence in Saudi Arabia is considered high, especially among children with reports from different cities ranging between 79.7% and 86.0% [5–7].

Dental caries begins as an incipient lesion that can be reversed by topical fluoride administered through oral hygiene products or professionally by dental care providers [8]. If left untreated, the carious process advances to a cavitated lesion, which may then include the pulp, causing pain, swelling, and eventually systemic symptoms [8]. Dental caries has devastating consequences on the child's general health and well-being. From pain, problems with mastication and speaking, poor nutrition, and poor school performance [9], to the psychological impact and low self-esteem [10]. In the same context the emotional, physical, and financial burden on the parents and family cannot be overlooked [11]. The possible consequences of not seeking preventive measures and early dental interventions put the child at risk of oral infections and pain that may require the use of general anesthesia to perform a comprehensive treatment [12, 13]. Parents must ensure that their children have adequate preventive care both at home and professionally as well as regular access to medical and dental services.

Fluoride varnish is a synthetic base or liquid resin that is administered topically and noninvasively a couple of times per year depending on the child's caries risk and was proven as an effective preventive measure against caries on both primary and permanent teeth [14]. FLV is one of the practical methods of topical fluoride administration to children due to its ease of application and tolerability when compared to other materials [15]. Many parental factors were found to influence children's oral health such as educational level of parents, employment [5] parent's oral health beliefs and attitudes alongside with supervised oral hygiene measures [16]. Parents' awareness of fluoride varnish in Saudi Arabia was found to be as low as 6.3% [17] alongside low utilization of dental services [17, 18]. In the same context, the cost of dental treatment is high and usually requires more than one visit. Noninvasive treatments, like fluoride application, aim to reduce biofilm cariogenicity through plaque control and rely heavily on patient compliance [19]. Increasing the acceptance and provision of preventive care requires changes in behavior, therefore exploring factors that influence individuals' views of preventive care may provide the opportunity to increase their awareness and encourage their compliance. Identifying reasons that cause refusal of preventive therapy is also important for clinicians to develop tailored educational approaches, as well as guide policymakers in planning health promotion campaigns. Therefore, this study aimed to investigate parental factors (demographics, source of knowledge, patterns of dental visits, perception of child's oral health and dental visits, and previous FLV experience) that influence knowledge, use, and acceptance to apply fluoride varnish in Eastern Saudi Arabia.

## 2. Methods

### 2.1. Study Design and Setting

This cross-sectional survey-based study was conducted in the main cities of the Eastern Province of Saudi Arabia namely Dhahran, Al Khobar, Al Dammam, Al Ahsa, Al Qatif, and Al Jubail (excluding villages and rural areas) in December 2021.

### 2.2. Study Participants

Parents (either mother or father) of children aged between 3 and 6 years residing in the Eastern Province and who agreed to participate in the study were included. Parents working in the dental field were excluded from the study.

### 2.3. Sample Size and Sampling Technique

The sample size was calculated through a Raosoft sample size online calculator [20]. Population was set as 200K (as sample size does not change much for bigger population) with a margin of error of 5% and estimated response distribution of 50% (FLV awareness) and a 95% confidence interval. The obtained sample size was 384 which was multiplied considering a design effect equal to 1.5 of cluster studies and the nonresponse rate; a total of 637 participants were recruited using a multistage sampling technique [21, 22]. This sample size was calculated to provide a framework within the population from which the participants will be recruited.

### 2.4. Data Collection Procedure

The survey was developed on Google forms and a QR code was generated and was then distributed online using social media mainly WhatsApp and Twitter. The QR code was shared with parents who were asked to scan and fill out the survey and were encouraged to share it with their friends and relatives. Reminders were not sent as all participants were not listed with the authors, but the survey was shared at multiple locations to capture maximum responses. The survey was distributed to parents in dental facilities, neighborhoods, and public parks in each of the Eastern Province's major cities (Dhahran, Al Khobar, Al Dammam, Al Ahsa, Al Qatif, and Al Jubail). Within the center of each city, first, we randomly selected a dental hospital and distributed the QR code, and when no more responses were received from hospitals, we then agreed on a well-known park (one of the mostly visited places by families) and a neighborhood (where either one of the team members lives or a personal connection) to reach out for parents. As the questionnaire was distributed online, we considered a lack or incomplete responses, and as such the questionnaire was distributed in all three sites within all cities at the same time. Parents accompanying children were approached randomly in each area by one of the research team members, and one caregiver (mother or father) with a child between the age of 3–6 years was requested to participate. The research team explained the study purpose, and the time needed to complete the survey and ensured the participants that the data was anonymous and would only be used for the research purposes.

### 2.5. Data Collection Tool

Data was collected using a validated self-administered questionnaire. The questionnaire was adopted and modified based on the previous literature [23–26]. The questionnaire consisted of four parts: demographics, knowledge, previous experience, and FV acceptance, and all were close-ended questions. Validation of the questionnaire was done in two ways. First, the face validity was evaluated by a group of experts (colleagues who are native speakers of Arabic and English) who reviewed the questionnaire and checked if there were any confusing or leading questions. Second, the questionnaire was pilot (both Arabic and English) tested before the beginning of the study with 20 parents. The responses from the pilot study were in line with the research objectives and interitem reliability was calculated using Cronbach alpha. The values of 0.638 and 0.71 for knowledge and previous experience were refereeing to the acceptable range for the questionnaire. None of the participants reported difficulty with the questions or needed any further explanation after distributing the questionnaire.

### 2.6. Demographics

This section asked about: (1) gender of the parent (male or female). (2) Parent's age (categorized as below 25, from 25 to 30 years, from 31 to 40 years, from 41 to 50 years, and above 50 years). (3) Nationality of the parents (Saudi and non-Saudi). (4) Parent's educational level (categorized as no education, school education/diploma, university degree, and above). (5) Family income (categorized as less than 5000 Saudi riyals (SR)/month, between 5000 and 20000 SR/month, and more than 20000 SR/month). Based on the data published by the Statista Research Department [27], the average monthly income across all sectors and nationalities in Saudi Arabia in the first half of 2021 was 6.5K Saudi riyals, and the gross salary was estimated to range from 4,770 (minimum average) to 21,031 (highest average). We recategorized the income level into less than 5K (less than minimum), between 5 and 20K (within average salary), and more than 20K SAR (higher than the average salary). (6) Number of children (categorized as having one child, 2 or 3 children, or having more than 3 children). (7) The last question asked if one of the parents works in the dental field (answered as yes or no).

### 2.7. Assessment of Knowledge

Six questions were used to assess the parent's knowledge. The first question asked if parents have heard about FV before (answered as yes, no, not sure), and if they heard about FV, what their source of information was; parents can choose one or more options (media, Internet, dentist, friends, or others). The rest of the questions asked parents about the role/use of FV, eligibility for fluoride varnish, and how often should a child visit the dentist for fluoride therapy.

### 2.8. Previous Fluoride Varnish Experience

Parents were asked if (1) they have applied FV to their child/children before. If they answered yes, they were asked to describe their and their children's experiences. They were also asked if they were satisfied or not.

### 2.9. Fluoride Varnish Acceptance

The following three questions assessed the acceptance of the parents. (1) Will you apply it again for your child/children? (Yes or no) (2) Will you accept the application of fluoride varnish for your child? (Yes, no, or maybe) (3) Reason of refusal (if present) (I know what it is, and I refuse its application because it's harmful; I know what it is, and I refuse its application because it has no benefit; I do not know its benefits, it could be harmful; incapable financially; not available in my area).

The questionnaire was concluded by asking the parents (1) how they perceive their child's oral health, and they respond as good, acceptable, or poor. (2) If they find dental visits stressful (they answered as yes, no, or not sure). (3) If they do visit the dentist regularly (they answered as yes, no, or not sure).

### 2.10. Ethical Considerations

This study was approved by the Deanship of Scientific Research at Imam Abdulrahman bin Faisal University (IRB-2022—02–085). The survey was preceded by an explanation of the purpose of the study, the research team, and the time required to complete it. Participants were informed of the confidentiality and anonymity of their responses, as well as the importance of their voluntary participation. Informed consent was obtained from participants verbally and/or by reading the survey introduction and choosing to proceed with the questionnaire.

### 2.11. Statistical Analysis

Data was downloaded from Google forms as an Excel sheet, it was then refined and coded before importing it to SPSS (Version 24, IBM USA) for analysis. Descriptive statistics were presented using frequencies, and percentages, in the form of tables and figures where appropriate. As part of inferential statistics, the chi-square/exact Fisher's tests (where suitable) were employed to analyze the association between the different categorical variables. Univariate and multivariate logistics regression were performed to check possible associations of demographical factors with fluoride varnish (FV) acceptance by parents. Statistical significance was defined as a *P* value less than 0.05.

## 3. Results

A total of 623 parents responded to the survey with the majority 458 (73.5%) being females, Saudis 590 (94.7%), with middle-income level 332 (53.3%), had more than three children 370 (59.4%), and only 88 (14.1%) of the parents were working in the medical field. The majority of mothers aged between 41 and 50 years, 214 (34.3%) and 333 (53.5%) had higher education, while most fathers (285 (45.7%)) aged above fifty years with almost an equal degree of education (328 (52.6%)) (Table 1).

**Figure 1:**
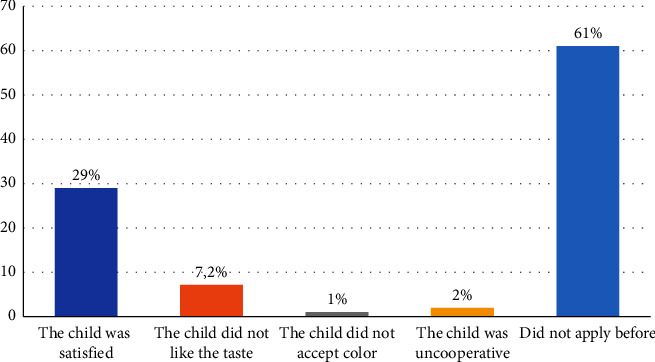
Parents' perceptions about fluoride varnish previous experience.

Of the participants, 338 (55%) of the parents had no previous knowledge about fluoride varnish.  Figure 1 shows that dentists were the main source of information among the study participants 145 (23%), followed by social media 74 (12%).  Table 2 shows the factors that influenced parental knowledge about fluoride varnish. Only 230 (36.9%) of the parents had good knowledge with females being the majority (80%), a difference that was statistically significant (*P*=0.003). Father's age (*P*=0.004), mothers' educational level (*P*=0.001), father's educational level (*P*=0.025), family income level (*P*=0.006), and being in the medical field (*P*=0.001) were all factors that significantly influenced parental knowledge about fluoride varnish.

**Figure 2:**
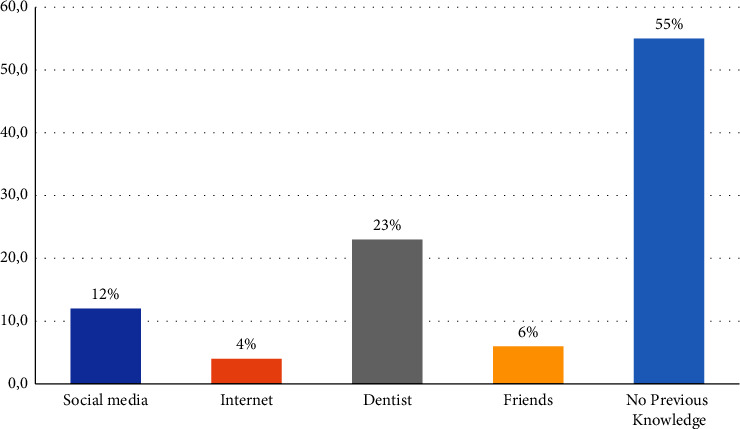
Sources of fluoride varnish knowledge as reported by parents.

Only 153 (24.6%) of the parents reported applying fluoride varnish to their children before.  Figure 2 shows parental experience with previous fluoride applications, and 29% reported that their children were satisfied with the fluoride varnish application.  Table 3 shows the factors associated with fluoride varnish's previous application among the study participants. Parental knowledge about fluoride varnish (its use, application, and dental visits) and willingness to apply it were factors that significantly influenced fluoride use among the study participants. Similarly, parental perceptions about their children's oral health were a significant influencing factor (*P*=0.014) among parents who perceived their children's oral health as good. Surprisingly, parental views of dental visits as a stressful experience significantly influenced fluoride varnish use among the study participants (*P*=0.014).

**Figure 3:**
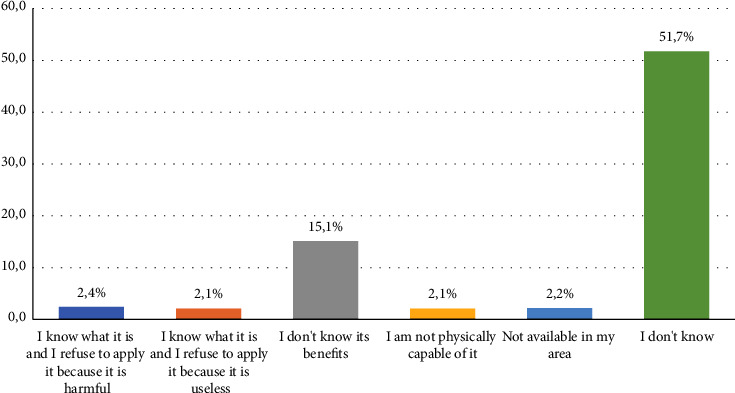
Reasons of refusing fluoride varnish application as reported by parents.

Figure 3 shows the reasons for refusing fluoride varnish application as reported by parents, 15.1% of parents reported not knowing the benefits of fluoride application as the reason for their refusal, while the majority (51.7%) refused the fluoride varnish application for no reason.  Table 4 shows the factors associated with the acceptance or rejection of fluoride varnish between both parents. An income more than the low level was a statistically significant factor that influenced the father's acceptance to apply fluoride varnish. On the other hand, age (mothers older than thirty years, *P*=0.02), educational level (educated mothers, *P*=0.017), and regular dental visits (mothers who did not visit the dentist regularly, *P*=0.0001) were factors that significantly influenced mothers' acceptance of applying fluoride varnish to their children. Some factors were significantly associated with both parents' preferences (acceptance or rejection). These included working in the medical field (working in the medical field, *P*=0.029 for father Vs *P*=0.001 for mother), perceiving dental visits as stressful (*P*=0.007 and *P*=0.024 for father and mother, respectively), source of knowledge (receiving FV information from dentists, *P*=0.0001 for both fathers and mothers), and overall FV knowledge *P*=0.0001 for both parents.

## 4. Discussion

This study highlights the low level of knowledge, use, and acceptance of fluoride varnish among parents in Eastern Saudi Arabia. There were more factors influencing mothers' acceptance of fluoride varnish application compared to fathers. Age, educational level, working in the medical field, perception about dental visits, patterns of dental visits, source of knowledge, and extent of knowledge were all factors that influenced a mother's decision to accept fluoride varnish. The study also highlights the crucial role dentists can play in changing the perspectives of parents and increasing the acceptance rate of fluoride varnish applications.

More than half of the parents in the current study had no previous knowledge about fluoride varnish. The poor level of knowledge observed in the current study about fluoride varnish is in line with multiple studies conducted elsewhere [23, 28–30]. Gender had a significant influence on knowledge in the current study, mothers were more knowledgeable about fluoride varnish compared to fathers. Although women always showed better results when it came to health knowledge and behaviors [31–33], reports about the role of gender with regard to FV knowledge were not uniform in previous studies, for example, Taravati and Lak [28], and Tsai et al. [34] reported similar findings to the current study while Tahani et al. [35] failed to link gender to the level of knowledge.

Socioeconomic and educational levels significantly affected the knowledge about fluoride varnish in the current study. Parents with higher educational degrees and socioeconomic status showed more knowledge about FV, this finding was supported by similar studies done previously [23, 24, 34, 35]. Lower educational and socioeconomic levels were found to be a predictor of poor oral health knowledge and poor oral hygiene habits [31, 36]. The relationship between higher educational and socioeconomic levels can be well explained through Maslow's hierarchy of needs [37], when people cannot secure their basic needs such as housing or daily expenses, then oral health literacy, regular dental visits, dental homes, and seeking preventive measures are understandably a secondary concern.

Parents who received FV information from their dentists had better knowledge and were more likely to accept the application of FV, in line with the results of Tahani et al. [35]. Although we found that the socioeconomic status influenced the level of knowledge in the current study, yet the previous work reported that individuals with dental insurance still avoid regular and preventive dental care suggesting the presence of additional determinants [38]. Health literacy is a proven mediator between socioeconomic determinants, health behavior, and oral health outcomes in a variety of communities [39]. Low health literacy has been linked to barriers to care and unfavorable oral health habits such as seeking preventive care [40].

Health information provided by care providers is valued by individuals and has a greater impact on improving oral health literacy and oral health behaviors [39]. According to research, it was found that individuals who get health information from health care providers are more likely to follow self-care precautions, prescriptions, and follow-up recommendations [41]. Dentists are encouraged to establish rapport with parents and use different communication methods in oral health education such as teach-back method, flyers, and visual aid materials [39] to simplify oral health information and instructions. Moreover, understanding the oral health literacy levels of parents is crucial for developing effective health educational materials and intervention programs to promote fluoride varnish use.

Fluoride use was also low in the current study as less than a quarter of parents reported applying it to their children before. Parents' awareness of the fluoride varnish and its benefits as a preventive intervention in the current study was low, a recent study used the economic principle of utility to explain patient preference and valuation for FV use [42]. Utility represents the improvement in well-being from treatment, and individuals are said to act in a manner that maximizes their utility [42]. Therefore, it is expected that the more knowledge the parents have about the benefits of FV, the more willing they will be to pay for fluoride varnish application.

Fluoride varnish use was greater among parents who perceived their children's oral health as good. Daly et al. explained the relation between parental perceptions and oral health behaviors as a circle, parents who perceived they did well in providing their children with proper medical and oral health care have children with better oral health which encouraged them more to sustain good oral health care for their children including the use of dental services and preventive care [43]. Being the primary decision makers in terms of their child's health, parental impressions of the care they provide are crucial since their decisions influence their child's well-being. It is as such important that dentists update parents during each dental visit about the improvements seen in their child's oral health status and appraise their efforts.

Again, the role of dentists is highlighted in the current study. We found that the use of fluoride varnish was more among parents who valued the importance of regular dental visits and was low among parents who perceived dental visits as stressful. Proper knowledge about the need for regular dental visits can facilitate preventive seeking behaviors [39] and will allow for early intervention and the use of less invasive and conservative treatments as such, leading to less stressful and painful dental visits.

The majority of parents and children who applied FV before were not satisfied with the experience which was significantly associated with fluoride varnish use. Factors for such low satisfaction levels may be also related to a lack of knowledge about the benefits of FLV. Similarly, the bitter taste and yellow discoloration can be unfavored by many parents. In addition, there are misconceptions circulated and promoted by the media. In a recent study, mothers reported receiving wrong and inconsistent information about fluoride application which resulted in them being confused and hesitant about fluoride benefits [40]^.^ Misconception and misinformation may influence patients' satisfaction with the care provided as well as treatment outcomes [41]. Having said so, FV satisfaction can further be improved by proper evaluation of parents' previous knowledge and trying to correct any misconceptions or concerns that parents have before applying FLV. It can also be beneficial if the dentist explains to the parents what to expect (bitter taste and discoloration) and the benefits the child will gain from such an application. Also, with the rapid innovations in the dental industry. It is expected that in the near future they can modify fluoride varnish products to overcome their bitter taste and transient discoloration. Qualitative studies are recommended to look closely at the information received through different media and suggest possible policies to control the type of information provided to the public.

Although we observed common factors that influenced FV acceptance among both parents, there were more factors influencing mothers' decisions compared to fathers. Regarding the gender effect, women were reported to care more about their health than their counterparts as well as the health of their children[44, 45]. The role of maternal socioeconomic characteristics and beliefs on their children's oral health is well documented in the literature [46]. In the current study, mothers' age, educational level, and employment (working in the medical field) were found to be associated with greater acceptance of FV application. Highly educated mothers and especially those with medical backgrounds will be better oriented about the consequences of dental caries and the available options for caries prevention. In the same context, younger aged mothers, regardless of their education level and background, are native-Internet users [44, 47]. Internet use for health concerns was observed more among females compared to males, especially those with children or expecting a child [45] and can therefore search for health information about the benefits, safety, and efficiency of FV and appraise its use as a preventive measure.

An interesting finding in the current study was that mothers who were not regular dental visitors and perceived dental visits as stressful had higher rates of FV acceptance, contradicting the findings by Alhareky et al., who found that mothers' dental anxiety was associated with an increase in caries prevalence [48]. Other factors, such as mothers' age, socioeconomic status, and educational level, may have acted as moderators, weakening the relationship between maternal dental anxiety and dental caries and encouraging anxious mothers to seek preventive care [49].

The source of FV knowledge (from dentists) and the level of knowledge (good knowledge) were significantly associated with the acceptance of FV application. In the current study, unawareness of benefits among parents was a major reason for FV refusal as well as concerns about its safety. Carpiano and Chi found that safety concerns and perceived disease severity were directly linked to fluoride application acceptance and refusal [50]. In Saudi Arabia, the prevalence of dental caries is high, and the cost of dental treatment is one of the major barriers to regular dental visits [51]. Therefore, dentists should provide detailed information about different preventive measures, namely, FV, given its effectiveness in the prevention of dental caries [52, 55]. It is also recommended that dentists clarify the consequences of dental caries on oral general health and children's well-being. Similarly, the use of presumptive approaches rather than participatory approaches by dental care providers was linked to parental refusal behaviors [50].

Parental denial/neglect of preventive care can become an issue, resulting in a higher disease burden for children and possibly higher health care expenses. Dental care providers can use parental characteristics (such as gender, educational level, attitudes, and perceptions) as identification tools in the clinic to predict those who are likely to decline FV application or preventive measures in general, therefore, allowing for well-tailored and specified educational interventions [50]. Although in the current paper we are encouraging the promotion of fluoride varnish application, there are certain factors that dentists need to consider and explain to parents. A recent systematic review reported that the prevalence of fluorosis with various degrees in Saudi Arabia ranged from 0 to 0.19, and that of esthetic fluorosis was around 0.07 to 0.76 [53]. Nowadays children can receive fluoride from multiple sources such as toothpaste, milk, juices, and professional applications [8, 15, 54, 56] in addition to fluoride levels in community water. As such dentists need to evaluate the benefits of FLV against the risks of dental fluorosis before recommending FLV to parents.

There are some limitations in the current study that we would like to acknowledge. The cross-sectional design of this study allows the establishment of associations rather than cause and effect. Participants for the current study were recruited only from the centers of the main cities and urban areas, we believe that the observed difference between fathers and mothers will be even more prominent in rural areas given the conservative nature of these locations. Also, there is a chance that selection bias may have occurred during the data collection and that some groups were not recruited. The large number of female participants compared to males may have influenced the observed associations in the current study. Moreover, the data was collected through close-ended questions, some parents had no reasons for FV refusal, and as such, it was beneficial if there were some open-ended questions so parents can elaborate more on their feelings and drives. Future studies should use mixed research methods to explore the factors behind the parental refusal of FV applications. It is also recommended that future studies look into psychological determinants of preventive care use.

Despite the limitations, this is the first study to explore the use and refusal of fluoride varnish among parents in Eastern Saudi Arabia. And although health behaviors are affected by different cultural contexts, we believe that the findings of this study can guide decision makers elsewhere as well.

## 5. Conclusion

The current study highlights the lack of knowledge and application of FLV among parents. More factors influenced the mother's decision to accept FV compared to the father's. Dentists played a major role in parental knowledge and FLV acceptance, as such it is recommended that dentists educate parents about the available preventive measures according to their children's risk assessment and dental problems. Parents should also be encouraged to share their concerns or doubts about dental treatments with their dentists to avoid any misconceptions. In a country with high caries prevalence, preventive programs such as FLV education and application are crucial given their efficiency, noninvasiveness, and cost-effectiveness. Educational campaigns, especially through social media, should be tailored and directed to mothers.

## Figures and Tables

**Table 1:** Background information of the study participants (*N* = 623).

Variables	Responses	*N* (%)
Gender	Male	165 (26.5)
	Female	458 (73.5)

Mother's age	Under 25 years	47 (7.5)
	25–30 years	84 (13.5)
	31–40 years	143 (23)
	41–50 years	214 (34.3)
	Over 50 years	135 (21.7)

Father's age	Under 25 years	3 (0.5)
	25–30 years	39 (6.3)
	31–40 years	125 (20.1)
	41–50 years	171 (27.4)
	Over 50 years	285 (45.7)

Nationality	Saudi	590 (94.7)
	Non-Saudi	33 (5.3)

Mother's educational level	Uneducated	53 (8.5)
	School diploma	237 (38)
	University education and above	333 (53.5)

Father's educational level	Uneducated	37 (5.9)
	School diploma	258 (41.4)
	University education and above	328 (52.6)

Family's income	Low-income level (≤5000 riyals per month)	67 (10.8)
	Middle income (lower middle and upper middle from 5000 to 20,000 riyals per month)	332 (53.3)
	High income (more than 20,000 riyals per month)	142 (22.8)
	Not sure	82 (13.2)

Parents work in the medical field	Yes	88 (14.1)
	No	535 (85.9)

Number of children	One child	88 (14.1)
	Two or three children	165 (26.5)
	More than three children	370 (59.4)

**Table 2:** Factors that influence parents' knowledge about fluoride varnish (*N* = 623).

Variables	Responses	Knowledge score category (%)		*P* value
		Poor knowledge *n* = 393	Good knowledge *n* = 230	
Gender	Male	30.3%	20.0%	**0.003** ^*∗*^
	Female	69.7%	80.0%	

Mother's age	Under 25 years	8.9%	5.2%	0.168
	25–30 years	12.7%	14.8%	
	31–40 years	20.6%	27.0%	
	41–50 years	34.9%	33.5%	
	Over 50 years	22.9%	19.6%	

Father's age	Under 25 years	0.3%	0.9%	**0.004** ^*∗*^
	25–30 years	6.9%	5.2%	
	31–40 years	18.6%	22.6%	
	41–50 years	23.4%	34.3%	
	Over 50 years	50.9%	37.0%	

Nationality	Saudi	94.4%	95.2%	0.406
	Non-Saudi	5.6%	4.8%	

Mother's educational level	Uneducated	10.4%	5.2%	**0.001** ^*∗*^
	School diploma	43.0%	29.6%	
	University education and above	46.6%	65.2%	

Father's educational level	Uneducated	7.4%	3.5%	**0.025** ^*∗*^
	School diploma	43.5%	37.8%	
	University education and above	49.1%	58.7%	

Family's income	Low-income level (≥5000 riyals per month)	12.5%	7.8%	**0.006** ^*∗*^
	Middle income (lower middle and upper middle from 5000 to 20,000 riyals per month)	53.9%	52.2%	
	High income (more than 20,000 riyals per month)	18.8%	29.6%	
	Not sure	14.8%	10.4%	

Parents work in the medical field	Yes	8.9%	23.0%	**0.001** ^*∗*^
	No	91.1%	77.0%	

Number of children	One child	15.8%	11.3%	0.253
	Two or three children	25.2%	28.7%	
	More than three children	59.0%	60.0%	

Source of knowledge	Social media	12.2%	11.3%	**0.001** ^*∗*^
	Internet	2.3%	7.4%	
	Dentist	8.4%	48.7%	
	Friends	4.8%	9.1%	
	No knowledge	72.3%	23.5%	

**Table 3:** Factors associated with fluoride varnish (FV) previous application/use among the study participants.

Knowledge about FV		Applied FV before		
		Yes (%)	No (%)	*P* value
Knowledge about the uses of fluoride varnish	Reducing tooth decay	39.9	60.1	**0.0001** ^*∗*^
	Reducing the sensitivity of teeth	19.6	80.4	
	Teeth whitening	7.2	92.8	
	Helps with dental growth	16.7	83.3	
	I don't know	6.1	93.9	

Knowledge about dentition eligible for varnish application	Temporary/milk teeth	35.1	64.9	**0.0001** ^*∗*^
	Permanent teeth	13.8	86.2	
	Both permanent and temporary	38.7	61.3	
	I don't know	7.2	92.8	

Knowledge about regular dental visits	Every 6 months	34.9	65.1	**0.0001** ^*∗*^
	Every 12 months	27.3	72.7	
	When there is a complaint	8.8	91.2	
	I don't know	11.4	88.6	

Child oral health as perceived by parents	Good	28.0	72.0	**0.014** ^*∗*^
	Acceptable	22.0	78.0	
	Bad	6.5	93.5	

Parents and child satisfied with the experience	Yes	77.9	22.1	**0.0001** ^*∗*^
	No	10.2	89.8	
	May be	11.5	88.5	

Willingness to apply it	Yes	3.6	96.4	**0.001** ^∗^
	No	46.4	53.6	
	May be	4.6	95.4	

Dental visits stressful	Yes	22.1	77.9	**0.014** ^*∗*^
	No	29.0	71.0	
	Not sure	14.5	85.5	

**Table 4:** Factors associated with the acceptance or rejection of fluoride varnish between fathers and mothers.

Study variables		Father's acceptance or rejection				Study variables			Mother's acceptance or rejection		
		Yes (%)	No (%)		*P* value				Yes (%)	No (%)	*P* value
Father's age	Under 25 years	100.0		0.0	0.399	Mother's age	Under 25 years		50.0	50.0	0.02^*∗*^
	25–30 years	33.3		66.7			25–30 years		43.3	56.7	
	31–40 years	50.0		50.0			31–40 years		65.6	34.4	
	41–50 years	55.2		44.8			41–50 years		55.6	44.4	
	Over 50 years	40.4		59.6			Over 50 years		45.5	54.5	

Nationality	Saudi	44.0		56.0	0.249	Nationality		Saudi	52.2	47.8	0.305
	Non-Saudi	66.7		33.3				Non-Saudi	59.3	40.7	

Father's educational level	Uneducated	30.8		69.2	0.443	Mother's educational level		Uneducated	54.2	45.8	0.017^*∗*^
	Diploma	43.1		56.9				Diploma	44.6	55.4	
	University education and above	48.8		51.3				University education and above	58.5	41.5	

Family's income	Low-income level (>5000 riyals per month)	20.0		80.0	0.037^*∗*^	Family's income		Low-income level (>5000 riyals per month)	51.1	48.9	0.069
	Middle income (lower middle and upper middle from 5000 to 20,000 riyals per month)	51.2		48.8				Middle income (lower middle and upper middle from 5000 to 20,000 riyals per month)	49.6	50.4	
	High income (more than 20,000 riyals per month)	50.0		50.0				High income (more than 20,000 riyals per month)	64.6	35.4	
	Not sure	29.4		70.6				Not sure	47.7	52.3	

Works in the medical field	Yes	65.2		34.8	0.029^*∗*^	Works in the medical field		Yes	73.8	26.2	0.001^*∗*^
	No	41.5		58.5				No	49.1	50.9	

The number of children	One child	34.8		65.2	0.393	The number of children		One child	52.3	47.7	0.553
	Two or three children	52.8		47.2				Two or three children	56.6	43.4	
	More than three children	44.3		55.7				More than three children	50.8	49.2	

Do you find dental visits stressful	Yes	64.4		35.6	0.007^*∗*^	Do you find dental visits stressful		Yes	60.1	39.9	0.024^∗^
	No	36.4		63.6				No	46.6	53.4	
	Not sure	42.9		57.1				Not sure	54.5	45.5	

Do you visit the dentist regularly?	Yes	48.9		51.1	0.096	Do you visit the dentist regularly?		Yes	52.5	47.5	0.0001^*∗*^
	No	48.4		51.6				No	61.0	39.0	
	Not sure	25.9		74.1				Not sure	19.6	80.4	

Source of FV knowledge	Social media	10.8		2.2	0.0001^*∗*^	Source of FV knowledge		Social media	12.0	16.1	0.0001^*∗*^
	Internet	2.7		1.1				Internet	4.6	5.5	
	Dentist	36.5		8.8				Dentist	36.1	10.6	
	Friends	5.4		2.2				Friends	10.8	3.7	
	No knowledge	44.6		85.7				No knowledge	36.5	64.1	

Overall knowledge	Poor knowledge	29.4		70.6	0.0001^*∗*^	Overall knowledge		Poor knowledge	38.3	61.7	0.0001^*∗*^
	Good knowledge	84.8		15.2				Good knowledge	73.9	26.1	

## Data Availability

The data can be provided by the principal investigator upon reasonable request.
